# Exposure to various abscission-promoting treatments suggests substantial ERF subfamily transcription factors involvement in the regulation of cassava leaf abscission

**DOI:** 10.1186/s12864-016-2845-5

**Published:** 2016-08-03

**Authors:** Wenbin Liao, Yayun Li, Yiling Yang, Gan Wang, Ming Peng

**Affiliations:** Institute of Tropical Bioscience and Biotechnology, Chinese Academy of Tropical Agricultural Sciences, Haikou, 571101 China

**Keywords:** Cassava abscission zone, AP2/ERF, Ethylene, Water-deficit stress

## Abstract

**Background:**

Cassava plants (*Manihot esculenta* Crantz) have obvious abscission zone (AZ) structures in their leaf pulvinus-petioles. Cassava leaf abscission can be triggered by either 17 days of water-deficit stress or 4 days of ethylene treatment. To date, little is known about cassava AP2/ERF factors, and less is known regarding their roles in regulating abscission zone development.

**Results:**

Here, the cassava and *Arabidopsis* AP2/ERF genes were compared, finding that the cassava genome contains approximately 1.54-fold more ERF subfamily than the *Arabidopsis* genome. Microarray analysis was used to identify the AP2/ERF genes that are expressed in cassava leaf pulvinus-petiole abscission zones by comparing the AP2/ERF gene expression profiles of ethylene- and water-deficit stress-induced leaf abscission. In total, 99 AP2/ERF genes were identified as expressed in AZs across six time points during both ethylene- and water-deficit stress-induced leaf abscission. Comparative expression profile analysis of similar SOTA (Self Organizing Tree Algorithm) clusters at six time points during ethylene- and water-deficit stress-induced leaf abscission demonstrated that 20 ERF subfamily genes had similar expression patterns in response to both treatments. GO (Gene Ontology) annotation confirmed that all 20 ERF subfamily genes participate in ethylene-mediated signalling. Analysis of the putative ERF promoter regions shown that the genes contained primarily ethylene- and stress-related cis-elements. Further analysis of ACC oxidase activity in AZs across six time points during abscission shown increased ethylene production in response to both ethylene and water-deficit stress; however, the difference was more dramatic for water-deficit stress. Finally, the expression ratios of 20 ERF subfamily genes were analysed in two cassava cultivars, ‘*KU50*’ and ‘*SC5*’, that exhibit different levels of leaf abscission when challenged with the same water-deficit stress. The analysis indicated that most of the ERF genes were expressed at higher levels in the precocious abscission ‘*KU50’* cultivar than in the delayed abscission ‘*SC5’* cultivar.

**Conclusion:**

Ccomparative analysis of both ethylene- and water-deficit stress-induced leaf abscission shown that the ERF subfamily functions in the regulation of cassava abscission zone development.

**Electronic supplementary material:**

The online version of this article (doi:10.1186/s12864-016-2845-5) contains supplementary material, which is available to authorized users.

## Background

Plant cellular and developmental processes are significantly affected by many environmental conditions, e.g. drought, cold, high salinity, flood, submergence, pathogen attack and hormone stress [1, 2], and the processes are regulated by various transcription factors (TFs) [3, 4]. The apetala2/ethylene response factor (AP2/ERF) gene family is one of the most important plant transcription factor families [5]. AP2/ERF genes contain one to two AP2/ERF domains comprising 60–70 conserved amino acid residues [5] and perform important regulatory functions in plant stress defence. In *Arabidopsis*, 145 AP2/ERF genes have been characterized and are divided among five families [6]: those containing two AP2 domains (AP2), one AP2 domain and an ethylene-responsive factor domain (ERF), one AP2 domain and a dehydration-responsive element-binding protein domain (DREB), one AP2 domain and one B3 domain (RAV), and the Soloists [5]. The AP2/ERF super-family has been recently studied in various plants, e.g. bamboo [5], grape [7], cucumber [8], rice [1], tomato [9], peach [10], sorghum [10], Chinese cabbage [11] and soybean [12].

The AP2/ERF family is known to have important functions in plant growth and development, as well as in abiotic and biotic stress responses [13]. AP2 subfamily members regulate seed abscission zone [14, 15], petal [5], ovule [5], and leaf epidermal cell identity [5], as well as flower development and seed growth [5, 13]. The ERF subfamily has specific functions in the regulation of abscission zone development [14, 16] and biotic stress response [5]. The DREB subfamily is involved in drought [12], cold [5], salinity [5], water-deficit [5], heat and osmotic stress response [5]. The RAV subfamily functions in the ethylene response [5]. Four AP2/ERF genes have been implicated in abscission: *SHA1* in rice [14, 15], *SlERF.B3* [17] and *SlERF52* [16] in tomato, and *FUF1* in *Arabidopsis* [18]. *SHA1* and *FUF1* both function in AZ development, whereas the two tomato genes function in AZ breakdown.

Cassava (*Manihot esculenta* Crantz) is the most important root crop in the world as more than 700 million people depend on cassava for staple food [19]. Cassava plants have obvious abscission zone structures in their leaf pulvinus-petioles [20–22], which facilitate their high drought tolerance as the abscission zones control the inflexible leaf abscission mechanism [22–24]. When subjected to prolonged adverse environmental conditions, cassava adapt to the new environment by shedding some old leaves, resulting in substantially reduced root yield in adverse environments [24]. When water becomes available, the crop can recover by rapidly forming new canopy leaves with substantially increased photosynthetic rates compared with unstressed crops, compensating for yield losses and producing final yields approaching those of well-watered crops [25]. Cassava plants require a flexible leaf abscission mechanism. Many abscission-related AP2/ERF genes have been identified in *Arabidopsis* [26], tomato [16], rice [15], melon [27] and apple [28, 29]; however, no studies have identified and characterized cassava abscission zone AP2/ERF super-family members.

This study surveyed the AP2/ERF family members using phylogenetic analysis of the 196 cassava AP2/ERF genes, and expression profile analysis indicated that 114 and 103 AP2/ERF genes were expressed during ethylene- and water-deficit stress-induced leaf abscission, respectively. Comparative analysis of ethylene- and water-deficit stress-induced leaf abscission suggested that the expression of 99 AP2/ERF genes in response both treatments resulted in leaf abscission. SOTA clustering indicated that AP2/ERF genes exhibited similar expression patterns during ethylene- and water-deficit stress-induced leaf abscission, and the putative promoters of the genes were examined for motifs. Additionally, ACC oxidase activities were measured at various time points for both treatments. The important AP2/ERF genes were further studied in two cassava cultivars that exhibit different levels of leaf abscission when subjected to the same stress. Together, the data suggest that ERF subfamily genes regulate the progression of cassava leaf abscission.

## Results

### Cassava AP2/ERF gene identification

One hundred and ninety-six putative AP2/ERF genes were identified from the *Manihot esculenta* genome (annotation v.4.1) that were predicted to include one or two complete AP2/ERF domains (Additional file 1: Table S1). The gene set represents approximately (196/34,151) 0.5739 % of the annotated genes in the cassava genome (34,151 genes), which is very similar to the proportion of *Arabidopsis* genes (0.5481 %), and greater than the proportion of poplar (0.4390 %), foxtail millet (0.4407 %) and rice (0.4315 %) genes [30]. Based on the similarity of the amino acid sequences with the *Arabidopsis thaliana* AP2/ERF protein family, twenty-nine genes were identified as encoding proteins containing two AP2-domains and thus assigned to the AP2 family. Seven genes were predicted to encode one AP2-domain and one B3-domain and were thus assigned to the RAV family. One hundred and fifty-five genes were found to encode proteins containing a single AP2/ERF domain, and the genes could be further classified into two groups based on the similarity of their amino acid sequences: one hundred genes were found to potentially encode ERF subfamily members, whereas fifty five genes were predicted to encode DREB subfamily members. Two genes were assigned to the Soloist family based on their similarity to the *Arabidopsis* Soloist (At4g13040) [31]. Three genes were also found to contain one single AP2 domain; however, their homologies were quite low compared with the other AP2/ERF factors and were therefore assigned to the others subfamily (Table 1).

**Table 1:** Comparison the AP2/ERF family of *M.esculenta* with *A.thaliana*

Plant classification		*M.esculenta*		*A.thaliana*	
	Group	Number	Percent	Number	Percent
DREB	A1	6	3.06	6	4.14
	A2	5	2.55	8	5.52
	A3	4	2.04	1	0.70
	A4	20	10.20	16	11.03
	A5	11	5.61	16	11.03
	A6	9	4.59	9	6.21
	Total	55	28.06	56	38.63
ERF	B1	18	9.18	15	10.34
	B2	5	2.55	5	3.44
	B3	30	15.31	18	12.41
	B4	10	5.10	7	4.83
	B5	7	3.57	8	5.52
	B6	30	15.31	12	8.28
	Total	100	51.02	65	44.82
AP2		29	14.80	17	11.71
RAV		7	3.57	6	4.14
Soloist		2	1.02	1	0.70
Others		3	1.53	0	0.00
Total		196		145	

### Phylogenetic reconstruction of the cassava AP2/ERF superfamily

**Fig. 1:**
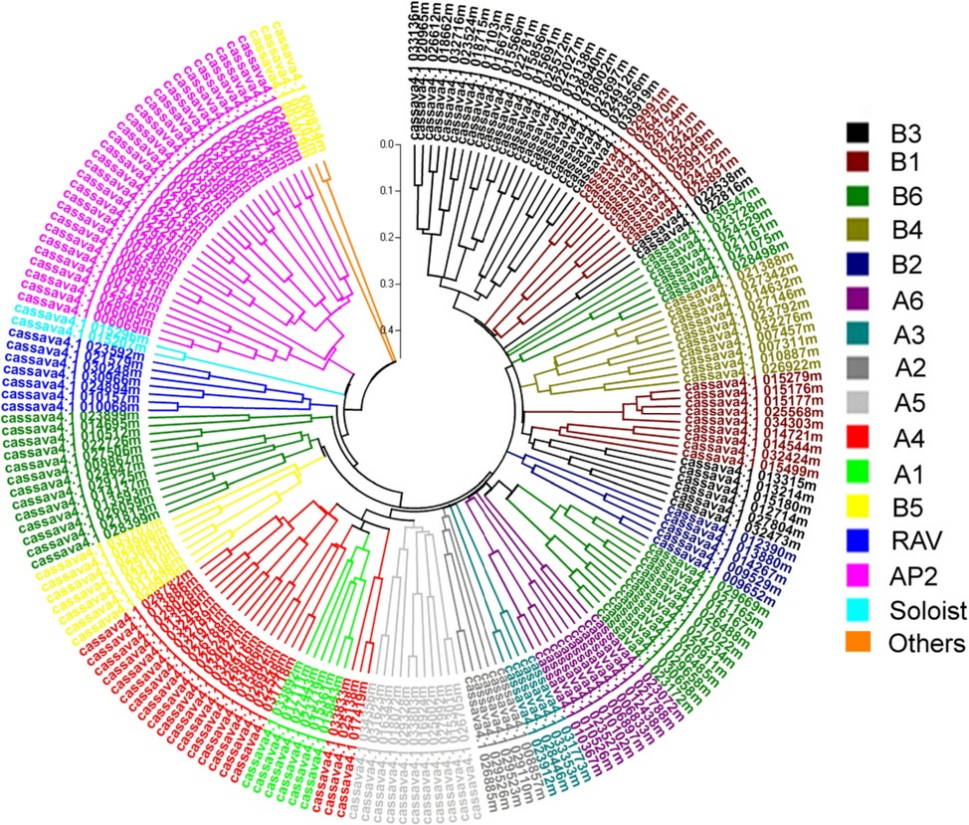
An unrooted phylogenetic tree of 196 cassava AP2/ERF transcription factors. Cassava AP2/ERF protein sequences were aligned using ClustalW, and the phylogenetic tree was constructed with MEGA 5.0 using the neighbour-joining method, based on the p-distance model with 1000 bootstrap replicates. Each subfamily is represented by a specific colour

To study the phylogenetic relationships among the cassava AP2/ERF super-family members, a phylogenetic tree were generated using 196 AP2/ERF amino acid sequences. The resulting phylogenetic tree contained 6 groups, termed AP2, RAV, DREB, ERF, Soloist and others. Members of the DREB group were classified into six subgroups: A1, A2, A3, A4, A5 and A6; similarly, the ERF genes were also classified into 6 subgroups: B1, B2, B3, B4, B5 and B6 (Fig. 1).

### Microarray identification of AP2/ERF genes that are expressed in cassava leaf pulvinus-petiole AZs during both ethylene- and water-deficit stress-induced leaf abscission

To compare the gene expression profiles during ethylene and water-deficit stress-induced cassava leaf abscission, leaf chlorophyll fluorescence (Fv/Fm) values [24] of 0.803, 0.765, 0.726, 0.697, 0.656 and 0.581 were represented as six time points (T1-T6). To verify the reliability and accuracy of differential gene expression profiling, AZs samples were collected with the same Fv/Fm values for both ethylene- and water-deficit stress-induced leaf abscission. Then used time course cassava whole genome microarray analysis to examine the AZ gene expression during ethylene- and water-deficit stress-induced leaf abscission using the T1 time point as a control.

To analyse the AZ gene expression profiles during ethylene- and water-deficit stress-induced leaf abscission, a cassava whole genome microarray (NimbleGen) containing 41,796 probe sets was constructed, representing the 34,151 JGI database transcripts (http://www.phytozome.net/cassava.php) and the 7645 GenBank transcripts. To identify differences in AP2/ERF gene expression, statistical analysis was used to screen the microarray data for genes that were differentially expressed in the treatment samples (T2-T6) compared with the T1 control. A stringent false discovery rate (FDR, <0.01) and log_2_T2-T6/T1 (≤0.5 or ≥ 2) values as thresholds were used to estimate the differences in gene expression during ethylene- and water-deficit stress-induced leaf abscission using three replicates. SOTA clustering performed to analyze the differences in gene expression profiles among the six leaf abscission time points, finding that FDR-corrected P values of <0.01 and significant changes in expression occurred at least one time point for each AP2/ERF gene expressed during ethylene- and water-deficit stress-induced leaf abscission.

**Fig. 2:**
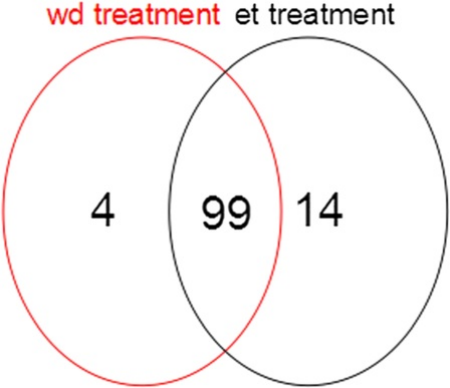
AP2/ERF genes shared between and unique to ethylene- and water-deficit stress-induced leaf abscission. wd: water-deficit stress; et: ethylene

**Fig. 3:**
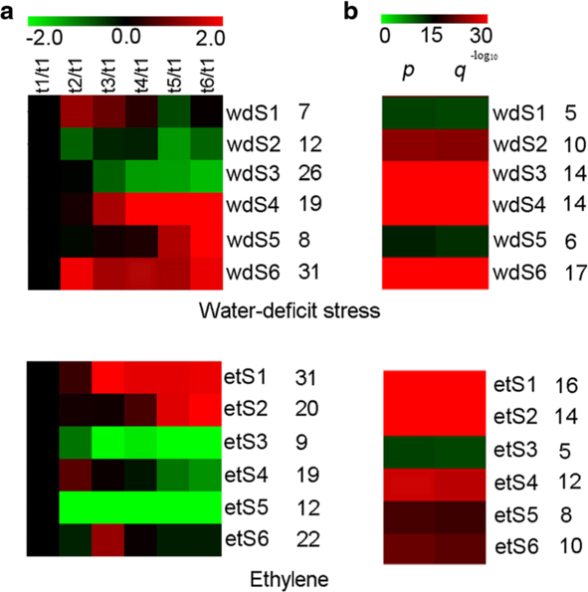
SOTA clustering showing the expression profiles of ethylene- and water-deficit stress-induced leaf abscission. **a** SOTA clustering identified six AP2/ERF gene expression clusters among the six leaf abscission time points (114 and 103 AP2/ERF genes for ethylene- and water-deficit stress-induced leaf abscission, respectively). The signals are indicated using a red-green colour scale, where red and green represent increased and reduced expression, respectively. **b** Ethylene signalling GO annotation enrichment of AP2/ERF genes expressed during both ethylene and water-deficit stress treatments among the six SOTA clusters

During ethylene-induced leaf abscission, 114 AP2/ERF genes were differentially expressed, whereas 103 AP2/ERF genes were differentially expressed during water-deficit stress-induced leaf abscission (Additional file 2: Table S2). Ninety-nine AP2/ERF genes were differentially expressed during ethylene- and water-deficit stress-induced leaf abscission, whereas 14 were differentially expressed only during ethylene-induced leaf abscission, and 4 were differentially expressed during water-deficit stress-induced leaf abscission (Fig. 2 and Additional file 2: Table S2). Of the AP2/ERF genes induced by water-deficit stress treatment, six SOTA clusters (wdS1-wdS6) could be separated into four groups of primary expression patterns (Fig. 3 and Additional file 3: Table S3). The first group, clusters wdS2 and wdS3, was down-regulated throughout the experimental period compared with the control. The second group, cluster wdS1, was up-regulated at the early and middle experimental time points, T2 and T3; whereas the third group, clusters wdS4 and wdS6, was up-regulated at the later experimental time points T4, T5 and T6; and the fourth group, cluster wdS5, was up-regulated at the latest experimental time points, T5 and T6.

The six ethylene-induced AP2/ERF gene SOTA clusters (etS1-etS6) were separated into five main expression patterns (Fig. 3 and Additional file 4: Table S4). The first group, clusters etS3 and etS5, was down-regulated throughout the experimental period compared with the control. The second group, cluster etS4, was up-regulated early, at T2; the third group, cluster etS6, was up-regulated at T3 during the middle of the experimental period; and the fourth group, cluster etS2, was up-regulated at the later experimental points T5 and T6. The fifth group, cluster etS1, was up-regulated throughout the experimental period but also exhibited the highest expression levels at T3 and T4 compared with the control.

### Comparison of AP2/ERF expression profiles between ethylene- and water-deficit stress-induced leaf abscission indicated that ERF subfamily genes are widely expressed in the cassava abscission zone

To identify the AP2/ERF genes that participate in both ethylene- and water-deficit stress-induced leaf abscission, the AP2/ERF expression profiles were compared in both treatments using SOTA clustering. Because the expression patterns of AP2/ERF genes were nearly identical between ethylene- and water-deficit stress-induced leaf abscission, the similar expression patterns at each time point were compared in both ethylene and water-deficit stress treatment.

The AP2/ERF genes that were up-regulated during the early experimental period (T2) in response to both treatments (Fig. 3a) were first examined. During ethylene treatment-induced leaf abscission, nineteen etS4 cluster AP2/ERF genes exhibited increased expression during the early stages of leaf abscission, and GO annotation indicated that 12 of the genes participate in ethylene-mediated signalling (GO:0009873) (Fig. 3b, Table 2, Additional file 5: Table S5). During water-deficit stress-induced leaf abscission, seven wdS1 cluster AP2/ERF genes exhibited increased expression during the early stages of leaf abscission, and GO annotation indicated that five of the genes participate in ethylene-mediated signalling (Fig. 3b, Table 2, Additional file 5: Table S5). Two genes, cassava4.1_025181m and cassava4.1_026705m, were expressed during the early stages of leaf abscission in response to both ethylene and water-deficit stress, and both participate in ethylene-mediated signalling (GO:0009873). Cassava4.1_025181m encodes CRF2, a member of the ethylene response factor (ERF) B-5 subfamily of ERF/AP2 transcription factors, it has the expression ratio (compared to T1 time-point) of 2.9774 in response to ethylene and 1.6156 to water-deficit stress respectively. Cassava4.1_026705m encodes DREB26, a member of the DREB A-5 subfamily of ERF/AP2 transcription factors, it has the expression ratio (compared to T1 time-point) of 4.3761 in response to ethylene and 2.1547 to water-deficit stress respectively.

**Table 2:** Gene count, p-Value and q-Value for AP2/ERF genes participated in ethylene-mediated signalling (GO:0009873) among six clusters in both ethylene and water-deficit stress treatment

Cluster	Gene count	p-Value	q-Value
et1	16	9.41E-37	5.65E-36
et2	14	6.68E-34	2.00E-33
et3	5	6.12E-12	1.22E-11
et4	12	1.17E-27	8.22E-27
et5	8	7.41E-20	2.22E-19
et6	10	5.85E-22	4.68E-21
wd1	5	6.12E-12	1.22E-11
wd2	10	4.88E-24	9.75E-24
wd3	14	6.42E-32	3.21E-31
wd4	14	8.37E-35	6.70E-34
wd5	6	7.47E-14	4.48E-13
wd6	17	1.52E-40	1.22E-39

At the middle stage of leaf abscission (T3), the etS6 and wdS1 groups (ethylene and water-deficit stress, respectively) exhibited similar expression patterns (Fig. 3a). Twenty-two AP2/ERF genes exhibited increased expression in response to ethylene treatment, and GO annotation indicated that ten participate in ethylene-mediated signalling (Fig. 3b, Table 2, Additional file 5: Table S5). At the T3 time point during water-deficit stress, seven AP2/ERF genes exhibited increased expression. Two genes, cassava4.1_015499m and cassava4.1_028940m, were expressed during both ethylene- and water-deficit stress-induced leaf abscission (Fig. 3b and Additional file 5: Table S5). Cassava4.1_015499m encodes ERF4, a member of the B-1 subfamily of ERF/AP2 transcription factors, it has the expression ratio (compared to T1 time-point) of 1.5052 in response to ethylene and 1.0759 to water-deficit stress respectively. Cassava4.1_028940m encodes ERF1, a member of the B-3 subfamily of ERF/AP2 transcription factors, it has the expression ratio (compared to T1 time-point) of 1.1519 in response to ethylene and 1.5544 to water-deficit stress respectively.

Later in leaf abscission (T5 and T6), etS2 and wdS5 (ethylene and water-deficit stress treatments, respectively) exhibited similar expression patterns (Fig. 3a). In response to ethylene treatment, nineteen AP2/ERF genes exhibited increased expression, and GO annotation indicated that fourteen participate in ethylene-mediated signalling (Fig. 3b, Table 2, Additional file 5: Table S5). In response to water-deficit treatment, eight AP2/ERF genes exhibited increased expression, and GO annotation indicated that six participate in ethylene-mediated signalling (Fig. 3b, Table 2, Additional file 5: Table S5). Comparative analysis indicated that three genes, cassava4.1_023899m, cassava4.1_027342m and cassava4.1_030658m, were expressed during both ethylene- and water-deficit stress-induced leaf abscission. Cassava4.1_023899m encodes CRF10, a member of the B-6 subfamily of ERF/AP2 transcription factors, it has the expression ratio (compared to T1 time-point) of 1.7343 in response to ethylene and 2.7688 to water-deficit stress respectively; cassava4.1_027342m encodes EBE, a member of the B-4 subfamily of ERF/AP2 transcription factors, it has the expression ratio (compared to T1 time-point) of 18.1761 in response to ethylene and 25.1308 to water-deficit stress respectively; and cassava4.1_030658m encodes ESE3, a member B-6 subfamily of ERF/AP2 transcription factors, it has the expression ratio (compared to T1 time-point) of 2.9389 in response to ethylene and 3.2621 to water-deficit stress respectively.

The etS1, wdS4 and wdS6 groups of genes were up-regulated throughout the experimental period (Fig. 3a). Thirty-one AP2/ERF genes exhibited this expression pattern in response to ethylene treatment, and GO annotation indicated that 16 participate in ethylene-mediated signalling (Fig. 3b, Table 2, Additional file 5: Table S5). Fifty AP2/ERF genes were up-regulated throughout the experimental period in response to water-deficit treatment, and GO annotation indicated that 31 participate in ethylene mediated-signalling (Fig. 3b, Table 2, Additional file 5: Table S5). Seventeen genes, cassava4.1_010068m, cassava4.1_010102m, cassava4.1_010512m, cassava4.1_014721m, cassava4.1_022726m, cassava4.1_023527m, cassava4.1_023697m, cassava4.1_007311m, cassava4.1_007457m, cassava4.1_013880m, cassava4.1_014267m, cassava4.1_014632m, cassava4.1_014695m, cassava4.1_015856m, cassava4.1_017103m, cassava4.1_022781m and cassava4.1_032424m were expressed during both ethylene- and water-deficit stress-induced leaf abscission. Cassava4.1_010068m encodes EDF1, a RAV transcription factor family member that contains AP2 and B3 binding domains, it has the expression ratio (compared to T1 time-point) of 1.734 in response to ethylene and 4.9585 to water-deficit stress respectively. Cassava4.1_010102m encodes RAP2.4, a DREB A-6 subfamily member of ERF/AP2 transcription factors, it has the expression ratio (compared to T1 time-point) of 1.1817 in response to ethylene and 1.5776 to water-deficit stress respectively. Cassava4.1_010512m encodes CRF11, a member of the ERF B-6 subfamily of ERF/AP2 transcription factors, it has the expression ratio (compared to T1 time-point) of 1.9445 in response to ethylene and 1.2128 to water-deficit stress respectively. Cassava4.1_014721m encodes ERF4, a member of the ERF B-1 subfamily of ERF/AP2 transcription factors, it has the expression ratio (compared to T1 time-point) of 3.6437 in response to ethylene and 2.9608 to water-deficit stress respectively. Cassava4.1_022726m encodes CRF11, a member of the ERF B-6 subfamily of ERF/AP2 transcription factors, it has the expression ratio (compared to T1 time-point) of 1.4961 in response to ethylene and 1.1308 to water-deficit stress respectively. Cassava4.1_023527m encodes RAP2.4, a member of the DREB A-6 subfamily of ERF/AP2 transcription factors, it has the expression ratio (compared to T1 time-point) of 1.5635 in response to ethylene and 1.2665 to water-deficit stress respectively. Cassava4.1_023697m encodes ERF12, a member of the ERF B-1 subfamily of ERF/AP2 transcription factors, it has the expression ratio (compared to T1 time-point) of 3.3057 in response to ethylene and 6.3907 to water-deficit stress respectively. Cassava4.1_007311m and cassava4.1_007457m encode a member of the ERF B-4 subfamily of ERF/AP2 transcription factors, cassava4.1_007311m has the expression ratio (compared to T1 time-point) of 1.4304 in response to ethylene and 132.6545 to water-deficit stress respectively; cassava4.1_007457m has the expression ratio (compared to T1 time-point) of 27.6667 in response to ethylene and 23.4243 to water-deficit stress respectively. Cassava4.1_013880m and cassava4.1_014267m both encode RAP2.3, a member of the ERF B-2 subfamily of plant-specific ERF/AP2 transcription factors, cassava4.1_013880m has the expression ratio (compared to T1 time-point) of 2.5673 in response to ethylene and 1.4501 to water-deficit stress respectively; cassava4.1_014267m has the expression ratio (compared to T1 time-point) of 2.6542 in response to ethylene and 3.6792 to water-deficit stress respectively. Cassava4.1_014632m encodes RAP2.6 L, a member of the ERF B-4 subfamily of ERF/AP2 transcription factors, it has the expression ratio (compared to T1 time-point) of 13.188 in response to ethylene and 20.2608 to water-deficit stress respectively. Cassava4.1_014695m encodes CRF9, a member of the ERF B-6 subfamily of ERF/AP2 transcription factors, it has the expression ratio (compared to T1 time-point) of 1.6535 in response to ethylene and 8.9521 to water-deficit stress respectively. Cassava4.1_015856m, cassava4.1_017103m and cassava4.1_022781m encode ERF1, a member of the ERF B-3 subfamily of ERF/AP2 transcription factors, cassava4.1_015856m has the expression ratio (compared to T1 time-point) of 29.6175 in response to ethylene and 9.8903 to water-deficit stress respectively; cassava4.1_017103m has the expression ratio (compared to T1 time-point) of 13.3218 in response to ethylene and 3.9972 to water-deficit stress respectively; cassava4.1_022781m has the expression ratio (compared to T1 time-point) of 17.3535 in response to ethylene and 6.7962 to water-deficit stress respectively. Cassava4.1_032424m encodes ERF9, a member of the ERF B-1 subfamily of ERF/AP2 transcription factors, it has the expression ratio (compared to T1 time-point) of 2.0164 in response to ethylene and 1.6363 to water-deficit stress respectively.

### Promoter motif prediction for ERF subfamily genes expressed during both ethylene- and water-deficit stress-induced leaf abscission

As described above, twenty-four genes were expressed in response to both ethylene and water-deficit stress, and twenty belonged to the ERF subfamily (Table 3). To further understand the potential functions of the genes in regulating abscission zone development, the promoters of the twenty ERF subfamily genes were analysed to identify cis-elements. For this analysis, 2 kbp sequences of putative promoter regions were examined for potential cis-regulatory elements that are responsive to water-deficit stress and ethylene [5]. Three drought stress response cis-elements, S000415, S000414 and S000176, as well as two ethylene response cis-elements, S000037 and S000457, were frequently identified within the promoter regions of the genes (Additional file 6). Many ERF genes contained more than twenty drought stress elements and more than five ethylene response elements, e.g. cassava4.1_025181m, cassava4.1_014721m, cassava4.1_022726m, cassava4.1_023697m, cassava4.1_007311m, cassava4.1_013880m, cassava4.1_014267m, cassava4.1_015856m, cassava4.1_017103m and cassava4.1_022781m (Additional file 6, Table 2).

**Table 3:** Twenty ERF Subfamily genes are widely expressed in the cassava abscission zone in both water-deficit stress and ethylene treatment

Gene ID	Best arabidopsis tair10 hit name	Subfamily	SOTA cluster in water-deficit stress	SOTA cluster in ethylene treatment	The highest expression in abscission	participate in ethylene-mediated signalling
cassava4.1_025181m	*CRF2*	ERF subfamily B-5	wdS1	etS4	early	Y
cassava4.1_015499m	*ERF4*	ERF subfamily B-1	wdS1	etS6	middle	Y
cassava4.1_028940m	*ERF1*	ERF subfamily B-3	wdS1	etS6	middle	Y
cassava4.1_023899m	*CRF10*	ERF subfamily B-6	wdS5	etS2	later	Y
cassava4.1_027342m	*NA*	ERF subfamily B-4	wdS5	etS2	later	Y
cassava4.1_030658m	*ESE3*	ERF subfamily B-6	wdS5	etS2	later	Y
cassava4.1_010512m	*CRF11*	ERF subfamily B-6	wdS6	etS1	Middle and later	Y
cassava4.1_014721m	*ERF4*	ERF subfamily B-1	wdS6	etS1	Middle and later	Y
cassava4.1_022726m	*CRF11*	ERF subfamily B-6	wdS6	etS1	Middle and later	Y
cassava4.1_023697m	*ERF12*	ERF subfamily B-1	wdS6	etS1	Middle and later	Y
cassava4.1_007311m	*NA*	ERF subfamily B-4	wdS4	etS1	Middle and later	Y
cassava4.1_007457m	*NA*	ERF subfamily B-4	wdS4	etS1	Middle and later	Y
cassava4.1_013880m	*RAP2.3*	ERF subfamily B-2	wdS4	etS1	Middle and later	Y
cassava4.1_014267m	*RAP2.3*	ERF subfamily B-2	wdS4	etS1	Middle and later	Y
cassava4.1_014632m	*RAP2.6 L*	ERF subfamily B-4	wdS4	etS1	Middle and later	Y
cassava4.1_014695m	*CRF9*	ERF subfamily B-6	wdS4	etS1	Middle and later	Y
cassava4.1_015856m	*ERF1B*	ERF subfamily B-3	wdS4	etS1	Middle and later	Y
cassava4.1_017103m	*ERF1B*	ERF subfamily B-3	wdS4	etS1	Middle and later	Y
cassava4.1_022781m	*ERF1B*	ERF subfamily B-3	wdS4	etS1	Middle and later	Y
cassava4.1_032424m	*ERF9*	ERF subfamily B-1	wdS4	etS1	Middle and later	Y

### ACC oxidase was up-regulated during ethylene- and water-deficit stress-induced leaf abscission

**Fig. 4:**
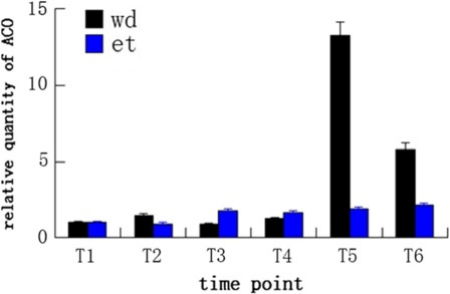
ACO measurements at six time points during ethylene and water-deficit stress treatments. wd: water-deficit stress; et: ethylene

GO annotation and promoter motif prediction of the twenty ERF subfamily genes expressed during ethylene and water-deficit stress treatments indicated functions in the ethylene response, suggesting that ethylene is an important factor in both ethylene- and water-deficit stress-induced leaf abscission. Measurements of ACC oxidase concentration (a key enzyme in ethylene biosynthesis) indicated that ACC oxidase activity gradually increased from T1 to T6. Furthermore, ACC oxidase activity levels were reduced during the early and middle stages of water-deficit stress-induced abscission compared with ethylene treatment. In contrast, ACC oxidase activities later increased sharply during water-deficit stress-induced abscission compared with ethylene treatment (Fig. 4). Together, the results indicate that ethylene was more concentrated in AZs during water-deficit stress than during ethylene treatment.

### Validation of comparative gene expression profiles of twenty ERF subfamily genes in two cassava cultivars with differing levels of leaf abscission

**Fig. 5:**
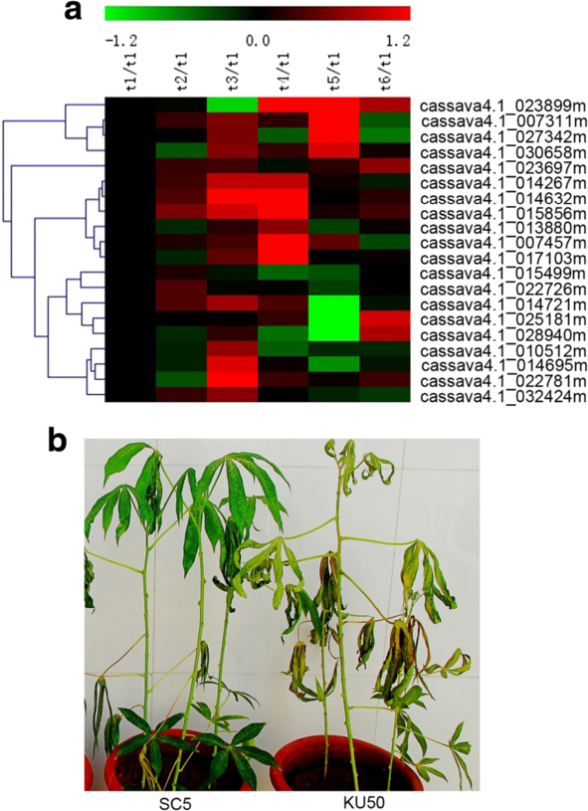
Identification of 20 ERF subfamily genes in two cassava cultivars (‘*KU50*’ and ‘*SC5*’) under water-deficit stress. **a** Heat map of real-time quantitative PCR (qRT-PCR) results for 20 ERF subfamily genes in two water-deficit stressed cassava cultivars; three biological and technical replicates were performed. **b** The water-deficit stress phenotype of two cassava cultivars, ‘*KU50*’ and ‘*SC5*’, at the middle stage of leaf abscission. Real-time PCR primer sequences are listed in Additional file 7: Table S7. The expression ratios for each ERF gene at each time point are presented first using T1 as a control for both ‘*KU50*’ and ‘*SC5*’, and then to cut ‘*KU50*’ expression ratios with those of ‘*SC5*’ at each time point. The heat map depicts the log2-transformed fold change values. The log_2_ (fold change values) and the colour scale are indicated above the heat map

To further understand the regulatory function of ERF subfamily genes in leaf abscission, their expression levels were examined in two cassava cultivars, ‘*KU50*’ and ‘*SC5*’. Compared to ‘*SC5*’ and other cultivars, ‘*KU50*’ is more easily drop off the leaves when met the same stress condition [19, 32, 33]. The expression ratios of the twenty ERF genes were compared between the cultivars at six time points during water-deficit stress, and clustered the ERF gene expression patterns using hierarchical clustering. The analysis indicated that most of the ERF genes (16) were more highly expressed at the middle and later time points (T3, T4, and T5) in the ‘*KU50*’ cultivar than in ‘*SC5*’. In particular, six ERF genes, cassava4.1_014267, cassava4.1_014632, cassava4.1_015856, cassava4.1_013880, cassava4.1_007457 and cassava4.1_017103 exhibited increased expression in ‘*KU50*’ compared with ‘*SC5*’ during the middle stages (Fig. 5a). At this stage, ‘*KU50*’ is more susceptible to dropping leaves than ‘*SC5*’ under the same conditions of water-deficit stress as all ‘*KU50*’ leaves, but few ‘*SC5*’ leaves, wither (Fig. 5b). Together, the data indicate that the middle stage is the key stage for determining leaf abscission.

## Discussion

### Comparative analysis of cassava and *Arabidopsis* AP2/ERF genes indicated a greater number of ERF subfamily members in the cassava genome than in the *Arabidopsis* genome

The AP2/ERF family is one of the most important subfamilies involved in plant development as well as abiotic and biotic stress response [2]. This study is the first to identify and characterize the AP2/ERF family in cassava. Here, one hundred and ninety-six cassava AP2/ERF genes were classified into 6 subfamilies (Table 1 and Fig. 1). Previously, Sakuma et al. (2002) reported the existence of 145 *Arabidopsis* AP2/ERF genes [6, 34]. We compared the cassava and *Arabidopsis* families and groups using the available complete genome sequences, finding more ERF and AP2 subfamily members within the cassava genome. Notably, the number of cassava ERF subfamily members (100) far exceeds that of the *Arabidopsis* genome (65) (Table 1 and Fig. 1). The number of DREB subfamily members is similar between the two genomes; however, the DREB subfamily sizes differ. Four *Arabidopsis* ERF groups, B1, B3, B4, and B6; and two DREB groups, A3 and A4, contained more members (Table 1). The number of cassava ERF subfamily members far outnumbers that in *Arabidopsis*, suggesting that this subfamily experienced a gene duplication event during cassava evolution. Gene duplication is considered to be the primary driving force of new gene functions [34]. In the study, more ERF genes were observed to participate in ethylene- and water-deficit stress-induced abscission, suggesting that the ERF subfamily plays more important functions in cassava plant development than in other subfamilies.

### ERF subfamily genes expressed at various time points function to regulate the progression of cassava leaf abscission

AP2/ERF genes have been shown to play crucial roles in various developmental processes, such as response to various stresses [13], phytohormone signalling [5, 13] and defence [1, 2]. Here, molecular methods were used attemptly to identify the key AP2/ERF genes that regulate cassava leaf abscission in response to ethylene and water-deficit stress. To maintain consistency between the two leaf abscission induction experiments, six Fv/Fm values were selected to serve as time points during leaf abscission for AZ sample collection prior to microarray analysis. To identify the AP2/ERF genes expressed during cassava leaf abscission in response to both treatments, the AP2/ERF gene expression profiles of pulvinus-petiole AZs were used for SOTA clustering. In total, 114 and 103 AP2/ERF genes were detectably expressed during ethylene- and water-deficit stress-induced leaf abscission, respectively (Fig. 2). Among the AP2/ERF genes, 99 were expressed during both ethylene- and water-deficit stress-induced leaf abscission (Additional file 2: Table S2), suggesting that similar genes regulate leaf abscission in response to the stresses. Comparative analysis of AP2/ERF gene expression profiles during ethylene- and water-deficit stress-induced leaf abscission resulted in the selection of 24 AP2/ERF genes distributed among the different time points, including 20 ERF subfamily genes, 3 DERB subfamily genes, and one RAV subfamily gene (Additional file 5: Table S5), suggesting that ERF subfamily genes play important roles in cassava abscission zone development. During early leaf abscission, high levels of CRF2 expression were detected (Fig. 3a and Additional file 2: Table S2, Additional file 3: Table S3, Additional file 4: Table S4, Additional file 5: Table S5) that mediate the cytokinin response [35], indicating that cassava plant maintained growth and development during the early stages of ethylene and water-deficit stress treatment. Abscission does not occur during the early stages. CRF2 expression decreased during the middle and late stages of abscission, suggesting that abscission occurs within AZs at the stages. The reduced expression this gene type in response to cytokinin may indicate the start of abscission.

During the middle of abscission, *ERF4* expression was high (Fig. 3a and Additional file 2: Table S2, Additional file 3: Table S3, Additional file 4: Table S4, Additional file 5: Table S5). *ERF4* was shown to direct leaf senescence progression, and transgenic *Arabidopsis* plants with increased *AtERF4* expression exhibited precocious leaf senescence [36]. Gene expression and chromatin immunoprecipitation assays suggested that this gene is involved in regulating the expression of many genes that are involved in leaf senescence progression [36]. High *ERF4* expression during the middle stages of cassava abscission suggested that senescence occurred in AZ cells at this stage and that AZ cells underwent precocious senescence in response to external stresses that induced *ERF4* expression [36].

Later in abscission, high *ESE3* expression levels were detected (Fig. 3a and Additional file 2: Table S2, Additional file 3: Table S3, Additional file 4: Table S4, Additional file 5: Table S5). *ESE3* has been suggested to regulate stamen abscission zone development by communicating with components of the HAE pathway within the ethylene-dependent pathway [37]. From this point, both ethylene and water-deficit stress treatments regulate leaf abscission via the ethylene-dependent pathway. The ACC oxidase activity measurements also confirmed that the ethylene contents of AZs increased during ethylene- and water-deficit stress-induced abscission, although their ethylene contents increased sharply during water-deficit stress treatment (Fig. 4). The data suggest that ethylene is a primary factor that contributes to ethylene- and water-deficit stress-induced cassava leaf abscission.

Seventeen AP2/ERF genes were highly expressed between T2 and T6 in response to both ethylene and water-deficit stress treatment (Fig. 3a and Additional file 2: Table S2, Additional file 3: Table S3, Additional file 4: Table S4, Additional file 5: Table S5). The genes that exhibited the expression pattern primarily contributed to cassava leaf abscission. High levels of *EDF1* expression at this stage have been reported to promote flower senescence and abscission, as well as activate senescence-associated genes downstream in the ethylene response [18]. *Rap2.4a* was also highly expressed at this stage; this gene is predicted to act in reactive oxygen species (ROS) signalling by controlling nuclear expression of 2-Cys peroxiredoxin A and other chloroplast antioxidant enzymes. In the previous study, ROS were proved to play an important role in the regulation of cassava leaf abscission [22]; furthermore, ROS are related to ethylene and water-deficit stress [38]. High expression levels of another *ERF4* gene were also detected at this stage. *RAP2.4* has been implicated in the abiotic stress response, acting to regulate multiple aquaporin genes that were also highly expressed in response to ethylene and water-deficit stress treatments, suggested that water homeostasis is disrupted during cassava leaf abscission [39]. Two *RAP2.3* genes, cassava4.1_013880m and cassava4.1_014267m, were also highly expressed at this stage and have been shown to participate in the responses to low oxygen, oxidative and osmotic stress [40]. GO annotation also suggested this gene functions to regulate cell death (Additional file 3: Table S3 and Additional file 4: S4), and comparison of their expression ratios between ‘*KU50*’ and ‘*SC5*’ confirmed increased expression in the ‘*KU50*’ cassava cultivar that exhibited precocious abscission when met the same stress (Fig. 5a). *RAP2.6 L* has been reported to induce premature senescence by increasing stomatal closure more than the high antioxidant enzyme activity levels that are also detected at that stage. The gene has also been reported to participate in the jasmonic, salicylic, and abscisic acid pathways as well as the ethylene pathway, indicated that many phytohormone pathways are involved in cassava leaf abscission [41]. Furthermore, increased expression levels were also observed in the precocious abscission ‘*KU50*’ cultivar compared with ‘*SC5*’ cultivar subjected to the same stress (Fig. 5a). Two *ERF1* genes, cassava4.1_015856 and cassava4.1_017103m, are highly expressed at that stage, and this gene has been shown to have early and transient oxidative activity during plant responses to osmotic, ionic, redox and hormonal signalling [42]. Comparison of the ‘*KU50*’ and ‘*SC5*’ *ERF1* expression ratios further confirmed increased expression in the precocious abscission ‘*KU50*’ cultivar when subjected to the same stress (Fig. 5a). Together, the data suggest that many signalling pathways are involved in cassava leaf abscission, e.g. hormonal, ROS, water stress, programmed cell death (PCD), and osmotic signalling.

### The downstream genes of six ERF genes expressed at higher levels in the precocious abscission ‘*KU50*’ cultivar

Since six ERF genes, cassava4.1_014267 (*RAP2.3*), cassava4.1_014632 (*RAP2.6 L*), cassava4.1_015856 (*ERF1*), cassava4.1_013880 (*RAP2.3*), cassava4.1_007457 (*ERF-B 4*) and cassava4.1_017103 (*ERF1*), expressed at higher levels in the precocious abscission ‘*KU50*’ cultivar than in the delayed abscission ‘*SC5*’ cultivar, suggested the six ERF genes play important regulation roles on abscission when suffered form stress. The analyses of the putative downstream genes of the six genes were examined. *Glutathione S-transferase 6* (*GST6*) can be regulated by *RAP2.3,* glutathione S-transferase (GST; EC 2.5.1.18) proved to play major roles in oxidative stress metabolism, the levels of GST, can be induced by diverse environmental stimuli, were necessary to maintain cell redox homeostasis and protect plants against oxidative stress [43]. Overexpression *RAP2.3* in *Arabidopsis* have been proved to cause up-regulation of *GST6*, suggested *GST6* act downstream of the *RAP2.3* [43]. *PDF1.2*, a plant defensin gene, related to ROS detoxification, was increased in *Arabidopsis* lines overexpressing *RAP2.3* and *ERF1* [43]. Actually, *PDF1.2* is a typical downstream gene of the ethylene/jasmonic acid signaling, thus, it is likely that *ERF* promotes transcription of *PDF1.2* through in vivo binding to the GCC-box [43].

*WEAK ETHYLENE INSENSITIVE2/ANTHRANILATE SYNTHASE α1* (*WEI2/ASA1*), encoding a rate-limiting enzyme in tryptophan (Trp) biosynthesis, *ASA1* has proved to act downstream of *ERF1* [44]*.* ERF1 can bind to *ASA1* promoter and directly up-regulate *ASA1* expression [44]. Which also has been examined to have the function of leading to auxin accumulation and ethylene-induced inhibition of plant growth [44], indicated *ASA1* regulate the abscission zone separation by regulate the homeostasis of auxin and ethylene. In addition, *Arabidopsis nudix hydrolase 7* (*AtNUDT7*) also proved to act downstream of *ERF1* because ERF1 protein has been examined to bind to the GCC-box motif in the *AtNUDT7* promoter [45]. *AtNUDT7* plays an important role in regulating redox homeostasis during stress/defense signaling [45]. The early responsiveness of *AtNUDT7* provides a useful marker especially during oxidative cell death in plants [45], indicated *AtNUDT7* may participate in regulating abscission zone development by oxidative cell death pathway.

Many antioxidant enzymes, including APX1 (ascorbate peroxidase 1, EC 1.11.1.11) and FSD1 (Fe-superoxide dismutase 1, EC 1.15.1.1), and the water logging responsive gene ADH1 (alcohol dehydrogenase 1, EC 1.1.1.1), were proved to participate ROS regulation [46]. All these encoded genes of these antioxidant enzymes acted downstream of *RAP2.6 L, RAP2.6 L* overexpression caused significant increases in the transcripts of these antioxidant enzymes [46], suggesting these antioxidant enzymes participate to regulate abscission zone development by modify the antioxidant system of plants.

### The expression profiles of other known genes involved in cassava abscission zone development

Two most important breakthroughs were made in the abscission research, one of the most important breakthrough in abscission research was the identification of *INFLORESCENCE DEFICIENT IN ABSCISSION* (*IDA*) to regulate cell separation processes in *A. thaliana* [47]*.* The *ida* mutant proved to fail to undergo floral organ abscission, while overexpression of *IDA* lead to premature and ectopic abscission [48]. IDA mediates its effect through the two LEUCINE-RICH REPEAT RLKs (LRR-RLKs) HAESA (HAE) and HAESA-LIKE 2 (HSL2), as the double knockout *hae hsl2* is phenotypically similar to the *ida* mutant and overexpression of *IDA* is not able to rescue this phenotype [47–49]. Another breakthrough in abscission research was the identification of the *jointless* (*j*) mutant locus in tomato [50], which causes the plant to fail to develop pedicel AZs. The *j* locus was isolated by map-based cloning and the wild-type gene encodes a MADS-box transcription factor [51]. In tomato, three MADS-box genes, including *JOINTLESS, MACROCAYLYX*, and *SlMBP21*, have been shown to be implicated in development of the flower abscission zone [52], SVP family MADS-Box proteins have also been confirmed the function on abscission regulation, moreover, SVP family MADS-Box proteins have been proved to have the same function as *JOINTLESS* in abscission regulation, and discovered to interacted with *MACROCAYLYX*, and *SlMBP21,* indicating the key regulation role on abscission development [52].

**Fig. 6:**
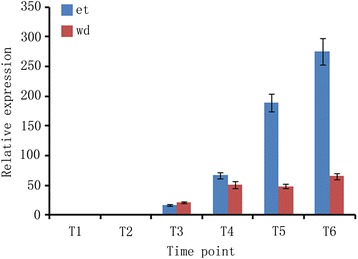
Identification of expression patterns of *IDA* gene under water-deficit stress and ethylene treatment by real-time quantitative PCR. wd: water-deficit stress; et: ethylene

In the cassava abscission zone, *IDA* expressed both in ethylene and water-deficit stress treatments, the expression patterns in both treatments were confirmed by qPCR in cassava abscission zones, the *IDA* expression levels had very low at the early stages of abscission in both ethylene and water-deficit stress treatments, however, the expression levels of *IDA* increased sharply at the later stages of abscission in both ethylene and water-deficit stress treatments, the highest expression ratios increased 275.3248 in ethylene treatment and 64.7765 in water-deficit stress treatment at T6 time point when compared to T1 time point respectively (Fig. 6).

**Fig. 7:**
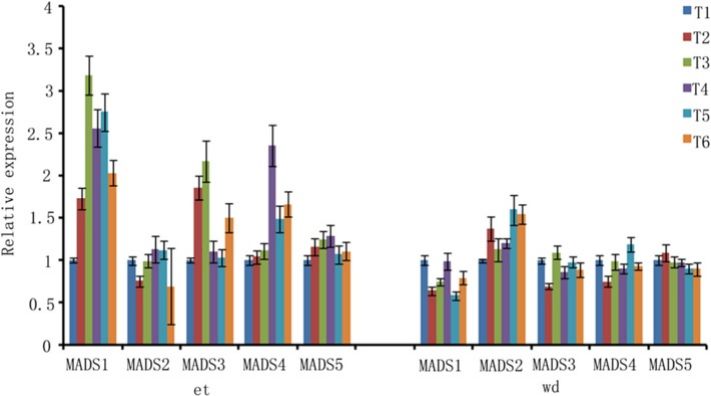
Identification of expression patterns of *MADS-box* genes under water-deficit stress and ethylene treatment by real-time quantitative PCR. wd: water-deficit stress; et: ethylene

Five MADS-Box proteins have been confirmed to express in abscission in both ethylene and water-deficit stress treatments (Fig. 7). Four MADS-box genes, *MADS1, MADS3, MADS4* and *MADS5* detected with high expression levels in ethylene treatment, the highest expression ratio of *MADS1* and *MADS3* appeared at T3 time point and had 3.2 and 2.2 times when compared with T1 time point (Fig. 7); the highest expression ratio of *MADS4* and *MADS5* appeared at T4 time point with 2.4 and 1.3 times when compared with T1 time point. One MADS-box gene, *MADS2* detected with high expression levels in water-deficit stress treatment, the expression ratio of *MADS2* had 1.6 times in T5 time point when compared with T1 time point (Fig. 7).

## Conclusions

More ERF subfamilies were found in the cassava genome than in the *Arabidopsis* genome. Comparative analysis of transcriptome expression profiles in response to ethylene and water-deficit stress indicated that most AP2/ERF genes were expressed in response to both treatments, suggested that the same AP2/ERF genes may function in leaf abscission in response to different treatments. Comparisons of AP2/ERF genes that were expressed at the same time points during abscission in response to both treatments indicated that 20 ERF subfamily genes were highly expressed during leaf abscission. Further analysis of promoter cis-elements and ACC oxidase activities confirmed that the ERF subfamily responds to ethylene and regulates cassava abscission zone development. The detection of high expression levels for most ERF subfamily genes in precocious abscission cassava cultivar verified the regulatory function of ERF subfamily genes in cassava abscission zone development.

## Methods

### Plant materials and treatments

*‘SC5’* and ‘*KU50’* cassava plants were grown as previously described [53]. In detail, cassava plants were planted in plastic pots at 28 °C under a 16 h light photoperiod (130 μmol · m^-2^ · s^-1^) for 3 months in the greenhouse. Three plants were planted in one pot, three pots repeated as a biological replicate. Three-month-old cassava plants with uniform growth statuses were chosen for ethylene and water-deficit stress treatments. Water-deficit stress and ethylene treatments were evaluated using the chlorophyll fluorescence parameter Fv/Fm. For ethylene treatments, leaves were sprayed with 100 μM ethylene, and plants were planted in a pot without water for water-deficit stress treatments. Fv/Fm values were used to select six time points for AZ sample collection.

Samples (approximately 1–2 mm) were cut from each pulvinus-petiole, including the AZs. AZs were collected from the middle of the cassava plants in each pots, three pots as a biological replicate, and repeated 3 times for each time points. AZs that were approximately 1–2 mm in size were frozen in liquid nitrogen for RNA extraction. RNA was purified using the plant RNA reagent from Invitrogen (Carlsbad, CA) to produce a pure and high-quality RNA preparation as indicated by spectroscopic and gel electrophoresis analysis.

### Identification of cassava AP2/ERF family genes

AP2/ERF genes were isolated from the cassava genome (JGI database, version 4.1) using annotation and BLAST. Cassava genome sequences that were suggested to contain an AP2 domain were isolated and identified as candidate AP2/ERF genes using the *Arabidopsis* genome as a reference [13]. The *Arabidopsis* AP2/ERF genes were identified using The *Arabidopsis* Information Resource (TAIR) [13]. Each putative cassava AP2/ERF gene was searched against the TAIR database using BLAST to ensure that no additional related genes were selected [13].

### Phylogenetic analysis

Phylogenetic analysis was performed using MEGA software, version 5, and the phylogenetic tree was constructed using neighbour-joining. The resulting tree was tested for reliability using bootstrapping with 1,000 replicates and amino acid p-distance parameters [54].

### Analysis of cassava ERF subfamily putative promoter region cis-elements

The putative promoter regions were analysed for cis-elements according to Wu et al. [5]. In detail, the 2 kbp putative promoter regions upstream of each ERF subfamily coding DNA sequence were examined for cis-elements using the PLACE website (http://www.dna.affrc.go.jp/PLACE/).

### The cassava whole genome microarray: design, hybridization, and data analysis

A time series of whole cassava genome microarray analyses based on the principle of the “loop design” was performed as previously described [55]. For either ethylene or drought treatment, 18 distinct AZ samples (three biological replicates at each of 6 time points) to be compared, the experimental design included 36 two-color microarray slides for both treatments, allowing three technical replicates of each sample to be observed. The cassava microarray was constructed as previously described [22, 55]. In detail, Two public databases were used for cassava microarray construction: the great majority of the ESTs originated from JGI database (http://www.phytozome.net/cassava.php) and the minority based on sequences from NCBI with E <1e-5. Custom-designed 60-mer nimblegen DNA microarrays were synthesized by maskless in situ photolithographic synthesis [22]. The fluorescent dye (Cy3-dCTP)-labeled cassava cDNA was produced as previously described using CapitalBio cRNA Amplification and Labeling Kit (CapitalBio). After completion of double-stranded cDNA (dsDNA) synthesis, the dsDNA products were purified using a PCR NucleoSpin Extract II Kit (MN). The resulting cRNA was labeled according to Nimblegen recommendations. The procedures of Array hybridization, washing, scanning and data analysis were performed at CapitalBio Corporation (Beijing, China) according to the NimbleGen’s Expression user’s guide. The expression data of probes were normalized using quantile normalization and expression data of genes were generated using the Robust Multichip Average (RMA) algorithm [22].

### Time course analysis

For comparative analysis, differences in gene expression between samples (T2-T6) and reference (T1) were identified using significant analysis of microarray software (SAM, version 3.02) [56]. Changes in gene expression exceeding a threshold of <0.5 or >2.0-fold change, with a Wilcoxon Rank-Sum test significance level of 0.01 (*P* < 0.01) and a false discovery rate (FDR) threshold of <1 % in the SAM output were considered to be differentially expressed. Time-dependent differentially expressed genes were classified with self organizing tree algorithm clustering (SOTA) using MeV 4.0 software [57].

### GO analysis

GO annotation of gene clusters was performed using BiNGO according to Maere et al. [58]. Significant GO categories were identified using a hypergeometric test with a significance threshold of 0.01 after a Benjamini and Hochberg FDR correction [59]. GO categories were classified by hierarchical clustering using MeV 4.0 software.

### Real-time RT-PCR validation of ERF subfamily gene expression in two cassava cultivars

For validation of comparative gene expression profiles of twenty ERF subfamily genes, ‘*KU50*’ cassava cultivar was chose as the control of the ‘*SC5*’ cassava cultivar, the two cassava cultivar that were cultivated widely in the south of China, ‘*KU50*’ was identified as a cassava cultivar with high-starch and different drought-resistance when compared to other cassava cultivars [19, 32, 33]. RNA from three independent biological samples were reverse transcribed and used for real-time qRT-PCR with SYBR Green I (Carlsbad, CA) detection on a STEP-ONE system. The real-time PCR primer sequences are listed in Additional file 7: Table S7. To avoid non-specific amplification from other AP2/ERF family genes, the primers were designed to span intron-exon boundaries or target the untranslated regions and the primer pairs were confirmed using the cassava genome database to ensure their specificity. The expression ratios of ERF genes at each time point are presented using T1 as a control first in both ‘*KU50*’ and ‘*SC5*’, and then to cut the ‘*KU50*’ expression ratios with those of ‘*SC5*’ at each time point.

### ACO measurements for ethylene and water-deficit stress treatments

ACO extractions and assays were performed according to He et al. [60]. Cassava leaf AZ tissue samples (3 g tissue, approximately 30 AZs) were collected at six time points from ethylene- or water-deficit stress-treated plants. The samples were ground in liquid nitrogen and used for extractions and measurements. The T1 sample ACO concentration served as a control.

## Abbreviations

AP2/ERF, APETALA2/ethylene response factor; AZs, abscission zones; FDR, false discovery rate; Fv/Fm, chlorophyll fluorescence; GO, gene ontology; SAM, significant analysis of microarray software; SOTA, self organizing tree algorithm.

## Additional files

Additional file 1: Table S1. Amino acid sequences of 196 cassava AP2/ERF proteins. (XLS 193 kb) Additional file 2: Table S2. Putative AP2/ERF genes expressed during ethylene- and water-deficit stress-induced leaf abscission. (XLS 1472 kb) Additional file 3: Table S3. SOTA clustering and annotation of AP2/ERF genes expressed in response to water-deficit stress treatment. (XLS 62 kb) Additional file 4: Table S4. SOTA clustering and annotation of AP2/ERF genes expressed in response to ethylene treatment. (XLS 67 kb) Additional file 5: Table S5. AP2/ERF genes that exhibited the same expression patterns during ethylene and water-deficit stress treatments. (XLS 19 kb) Additional file 6: Table S6. Summary of abiotic stress-inducible cis-elements in cassava ERF subfamily transcription factor promoter regions. (DOC 191 kb) Additional file 7: Table S7. Forward and reverse primers used for qRT-PCR analysis of ERF gene expression. (DOC 35 kb)
