# Supplementary material for: Exposure to various abscission-promoting treatments suggests substantial ERF subfamily transcription factors involvement in the regulation of cassava leaf abscission
Source: BMC Genomics. 2016 Aug 3;17:538. doi: 10.1186/s12864-016-2845-5 (PMC4973035; doi:10.1186/s12864-016-2845-5)
Supplement: Additional file 6: Table S6. — Summary of abiotic stress-inducible cis-elements in cassava ERF subfamily transcription factor promoter regions. (DOC 191 kb) [file 12864_2016_2845_MOESM6_ESM.doc]

**Additional file 6** Summary of abiotic-stress inducible cis-elements is in the promoter regions of ERF subfamily genes in cassava.

| **Abiotic**  **stress** | **Gene name**  ***cis*-element** | **motif sequence** | cassava4.1_025181m | cassava4.1_015499m | cassava4.1_028940m | cassava4.1_023899m | cassava4.1_027342m | cassava4.1_030658m |
| --- | --- | --- | --- | --- | --- | --- | --- | --- |
| Drought-stress | S000133 | CCACGTGG | 0 | 0 | 0 | 0 | 0 | 0 |
|  | S000153 | CCGAC | 0 | 0 | 0 | 0 | 0 | 0 |
|  | S000174 | CACATG | 0 | 1 | 1 | 0 | 2 | 3 |
|  | S000175 | CTAACCA | 0 | 0 | 0 | 1 | 0 | 1 |
|  | S000176 | CNGTTR | 10 | 2 | 3 | 4 | 4 | 1 |
|  | S000177 | TAACTG | 1 | 0 | 1 | 0 | 1 | 0 |
|  | S000402 | ACCGAC | 0 | 0 | 0 | 0 | 0 | 0 |
|  | S000408 | WAACCA | 4 | 2 | 0 | 2 | 4 | 2 |
|  | S000413 | CATGTG | 0 | 1 | 1 | 0 | 2 | 3 |
|  | S000414 | ACGTG | 10 | 4 | 1 | 1 | 0 | 1 |
|  | S000415 | ACGT | 18 | 6 | 8 | 6 | 2 | 6 |
|  | S000418 | RCCGAC | 0 | 0 | 0 | 0 | 0 | 0 |
|  | total |  | **33** | 16 | 15 | 14 | 15 | 17 |
| Wound-stress | S000244 | AACGTGT | 0 | 0 | 0 | 0 | 0 | 0 |
|  | S000444 | AGATCCAA | 0 | 0 | 0 | 0 | 0 | 0 |
|  | S000457 | TGACY | 5 | 3 | 1 | 0 | 1 | 4 |
|  | S000037 | AWTTCAAA | 1 | 1 | 2 | 2 | 1 | 3 |
|  | total |  | **6** | 4 | 3 | 2 | 2 | **7** |

| **Abiotic**  **stress** | **Gene name**  ***cis*-element** | **motif sequence** | cassava4.1_010512m | cassava4.1_014721m | cassava4.1_022726m | cassava4.1_023697m | cassava4.1_007311m | cassava4.1_007457m |
| --- | --- | --- | --- | --- | --- | --- | --- | --- |
| Drought-stress | S000133 | CCACGTGG | 0 | 0 | 0 | 2 | 0 | 0 |
|  | S000153 | CCGAC | 2 | 0 | 3 | 2 | 1 | 1 |
|  | S000174 | CACATG | 0 | 3 | 0 | 5 | 1 | 0 |
|  | S000175 | CTAACCA | 0 | 1 | 0 | 0 | 0 | 0 |
|  | S000176 | CNGTTR | 3 | 2 | 5 | 2 | 5 | 3 |
|  | S000177 | TAACTG | 0 | 0 | 2 | 0 | 1 | 0 |
|  | S000402 | ACCGAC | 0 | 0 | 1 | 0 | 1 | 1 |
|  | S000408 | WAACCA | 3 | 4 | 6 | 2 | 2 | 1 |
|  | S000413 | CATGTG | 0 | 3 | 0 | 5 | 1 | 0 |
|  | S000414 | ACGTG | 0 | 2 | 0 | 8 | 5 | 1 |
|  | S000415 | ACGT | 2 | 6 | 6 | 10 | 12 | 4 |
|  | S000418 | RCCGAC | 1 | 0 | 3 | 1 | 1 | 1 |
|  | total |  | 11 | **21** | **26** | **35** | **30** | 12 |
| Wound-stress | S000244 | AACGTGT | 0 | 0 | 0 | 0 | 0 | 0 |
|  | S000444 | AGATCCAA | 0 | 0 | 0 | 0 | 0 | 0 |
|  | S000457 | TGACY | 4 | 11 | 4 | 4 | 8 | 4 |
|  | S000037 | AWTTCAAA | 1 | 0 | 1 | 2 | 2 | 1 |
|  | total |  | **5** | **11** | 4 | **6** | **10** | **5** |

| **Abiotic**  **stress** | **Gene name**  ***cis*-element** | **motif sequence** | cassava4.1_013880m | cassava4.1_014267m | cassava4.1_014632m | cassava4.1_014695m | cassava4.1_015856m | cassava4.1_017103m |
| --- | --- | --- | --- | --- | --- | --- | --- | --- |
| Drought-stress | S000133 | CCACGTGG | 0 | 0 | 0 | 0 | 2 | 0 |
|  | S000153 | CCGAC | 1 | 0 | 2 | 0 | 0 | 0 |
|  | S000174 | CACATG | 1 | 1 | 4 | 1 | 1 | 1 |
|  | S000175 | CTAACCA | 0 | 0 | 0 | 0 | 0 | 0 |
|  | S000176 | CNGTTR | 6 | 5 | 3 | 3 | 9 | 3 |
|  | S000177 | TAACTG | 1 | 1 | 0 | 0 | 1 | 0 |
|  | S000402 | ACCGAC | 0 | 0 | 2 | 0 | 0 | 0 |
|  | S000408 | WAACCA | 3 | 5 | 0 | 2 | 2 | 3 |
|  | S000413 | CATGTG | 1 | 1 | 2 | 1 | 1 | 1 |
|  | S000414 | ACGTG | 5 | 4 | 0 | 1 | 2 | 3 |
|  | S000415 | ACGT | 16 | 14 | 0 | 4 | 4 | 9 |
|  | S000418 | RCCGAC | 1 | 0 | 2 | 0 | 0 | 0 |
|  | total |  | **35** | **31** | 15 | 12 | **22** | **20** |
| Wound-stress | S000244 | AACGTGT | 0 | 0 | 0 | 0 | 0 | 0 |
|  | S000444 | AGATCCAA | 0 | 0 | 1 | 0 | 0 | 0 |
|  | S000457 | TGACY | 4 | 3 | 6 | 0 | 5 | 9 |
|  | S000037 | AWTTCAAA | 1 | 2 | 2 | 3 | 1 | 2 |
|  | total |  | **5** | **5** | **9** | 3 | **6** | **11** |

| **Abiotic**  **stress** | **Gene name**  ***cis*-elem**  **ent** | **motif sequence** | cassava4.1_022781m | cassava4.1_032424m |  |  |  |  |
| --- | --- | --- | --- | --- | --- | --- | --- | --- |
| Drought-stress | S000133 | CCACGTGG | 0 | 0 |  |  |  |  |
|  | S000153 | CCGAC | 0 | 1 |  |  |  |  |
|  | S000174 | CACATG | 2 | 0 |  |  |  |  |
|  | S000175 | CTAACCA | 0 | 0 |  |  |  |  |
|  | S000176 | CNGTTR | 4 | 1 |  |  |  |  |
|  | S000177 | TAACTG | 1 | 0 |  |  |  |  |
|  | S000402 | ACCGAC | 0 | 0 |  |  |  |  |
|  | S000408 | WAACCA | 3 | 1 |  |  |  |  |
|  | S000413 | CATGTG | 2 | 0 |  |  |  |  |
|  | S000414 | ACGTG | 5 | 1 |  |  |  |  |
|  | S000415 | ACGT | 10 | 2 |  |  |  |  |
|  | S000418 | RCCGAC | 0 | 0 |  |  |  |  |
|  | total |  | **27** | **6** |  |  |  |  |
| Wound-stress | S000244 | AACGTGT | 0 | 0 |  |  |  |  |
|  | S000444 | AGATCCAA | 0 | 0 |  |  |  |  |
|  | S000457 | TGACY | 5 | 6 |  |  |  |  |
|  | S000037 | AWTTCAAA | 1 | 2 |  |  |  |  |
|  | total |  | **6** | **8** |  |  |  |  |
