# Supplementary material for: Exposure to various abscission-promoting treatments suggests substantial ERF subfamily transcription factors involvement in the regulation of cassava leaf abscission
Source: BMC Genomics. 2016 Aug 3;17:538. doi: 10.1186/s12864-016-2845-5 (PMC4973035; doi:10.1186/s12864-016-2845-5)
Supplement: Additional file 7: Table S7. — Forward and reverse primers used for qRT-PCR analysis of ERF gene expression. (DOC 35 kb) [file 12864_2016_2845_MOESM7_ESM.doc]

Additional file 7 Forward and reverse primers used in qRT-PCR for ERF genes expression analyses

| **Gene ID** | **Primer Designation** | **Sequences** |
| --- | --- | --- |
| cassava4.1_025181m | Forward  Reverse | TGCTATTTGGAGAAGCAGTGGGCC  TAGCCGCGTTGTCATAAACCATTGC |
| cassava4.1_015499m | Forward  Reverse | GTGTTCAGAGTGATTCAGATTCTTC  CTCCTGTCTCGACTGGAAATTGAG |
| cassava4.1_028940m | Forward  Reverse | CCAAGCTGAGTTGGGAAGGGATAG  AATTGGTGGTAAAGTTGTCCACTTAG |
| cassava4.1_023899m | Forward  Reverse | TGGAGTGTTGGATCTTCCGAAC  CATACTATCACTATCTGTGATTC |
| cassava4.1_027342m | Forward  Reverse | TTCAAGATCAAGGGAACACTAGGAG  GAGCAGCTTCGTCATAAGCAAGTGC |
| cassava4.1_030658m | Forward  Reverse | CATCCTTTACTGAAGACAAGAAT  TGAAGAAGATGATTGTGGCTCA |
| cassava4.1_010512m | Forward  Reverse | TTGCAGGATAGACAGTGATGAACC  CTCAAGAACCCATCTTCAGTTTCT |
| cassava4.1_014721m | Forward  Reverse | CTCATCCTATGTAGCTTTCATTGG  ACCTTTGATATGTCCACCGTCCAT |
| cassava4.1_022726m | Forward  Reverse | GTTAGACAGTGATGGACCAAGCGA  ACTCCCGAGATTAGTCCTTGCTGC |
| cassava4.1_023697m | Forward  Reverse | CGGATACTGGTGTTCAGCCGT  GATTTGATGTTACTCTTACTCCC |
| cassava4.1_007311m | Forward  Reverse | TCTGGTGCAACTGTAAAAGAAGAG  TCTAACTCCTCTGTATCTTCTTCT |
| cassava4.1_007457m | Forward  Reverse | TCTGGTGCAACTCTAACAGAAGAGC  CCACGGCCTTTGTCTAACTCCTCT |
| cassava4.1_013880m | Forward  Reverse | TCAGCCAAGTGGCCATCGGCAG  GATGCGCTTGGCTGCCTCATCG |
| cassava4.1_014267m | Forward  Reverse | GCTTAACCAAGTGGAGAAGACTGAG  GAACACCCTTGTGTGGGTCTCTGAT |
| cassava4.1_014632m | Forward  Reverse | CCAAGATCAAGATAATACAAGGAG  TCGAAAGTACCGAGCCAGACTCGT |
| cassava4.1_014695m | Forward  Reverse | TGCTGAGTGGTGATTATTGCTCAGA  CCAAAGCCTCCTATAATTCCTAACT |
| cassava4.1_015856m | Forward  Reverse | GATGAGCAGAAGCAAGAGAGAGAG  AGAGAAGTGGTCATCGAGATCACA |
| cassava4.1_017103m | Forward  Reverse | CAGAGTCGGAGAGCAGCACTACTG  CAATAGTTCTGGCTCTATCTTGAC |
| cassava4.1_022781m | Forward  Reverse | TTGGAAGACTTAGGTGCTGATTAC  GATCATACCCATCCACCCATATTC |
| cassava4.1_032424m | Forward  Reverse | GAGTGACTCGGATTCGTCATCTGTG  CCAGAACTCATCCTAAATTATCATC |
